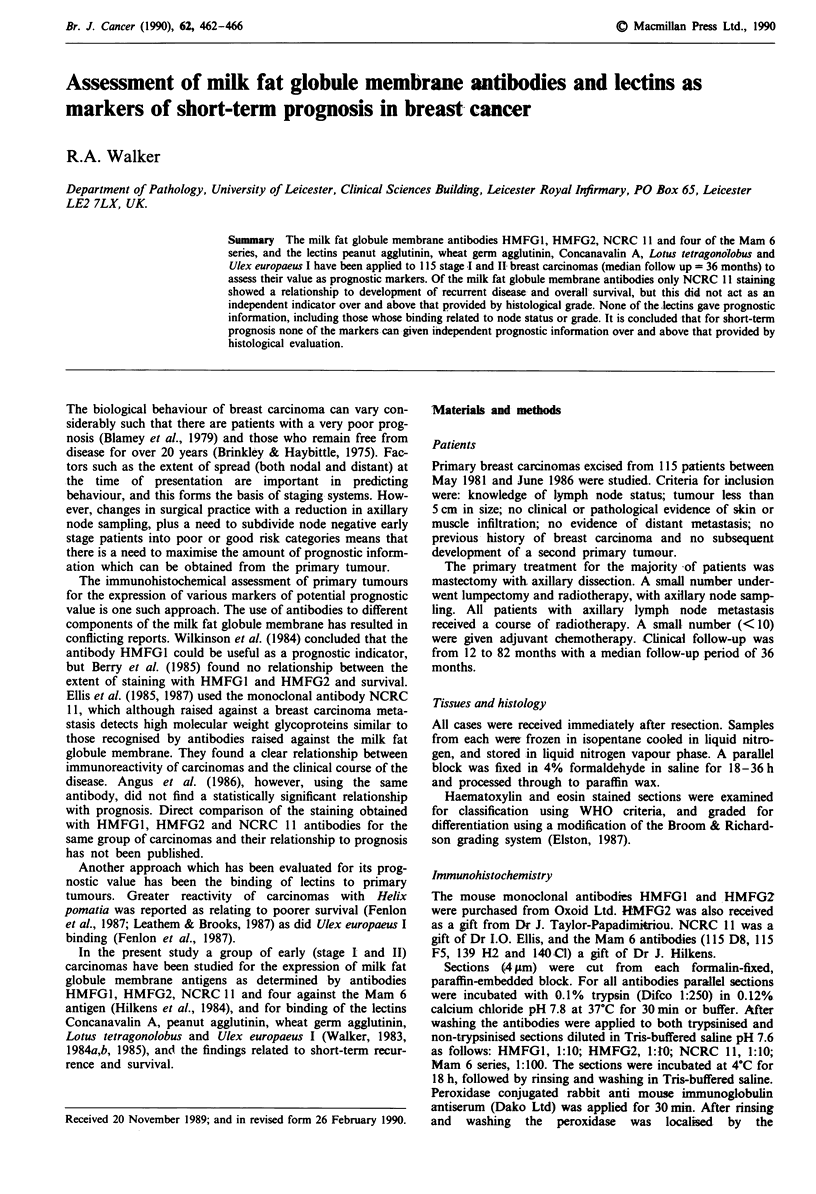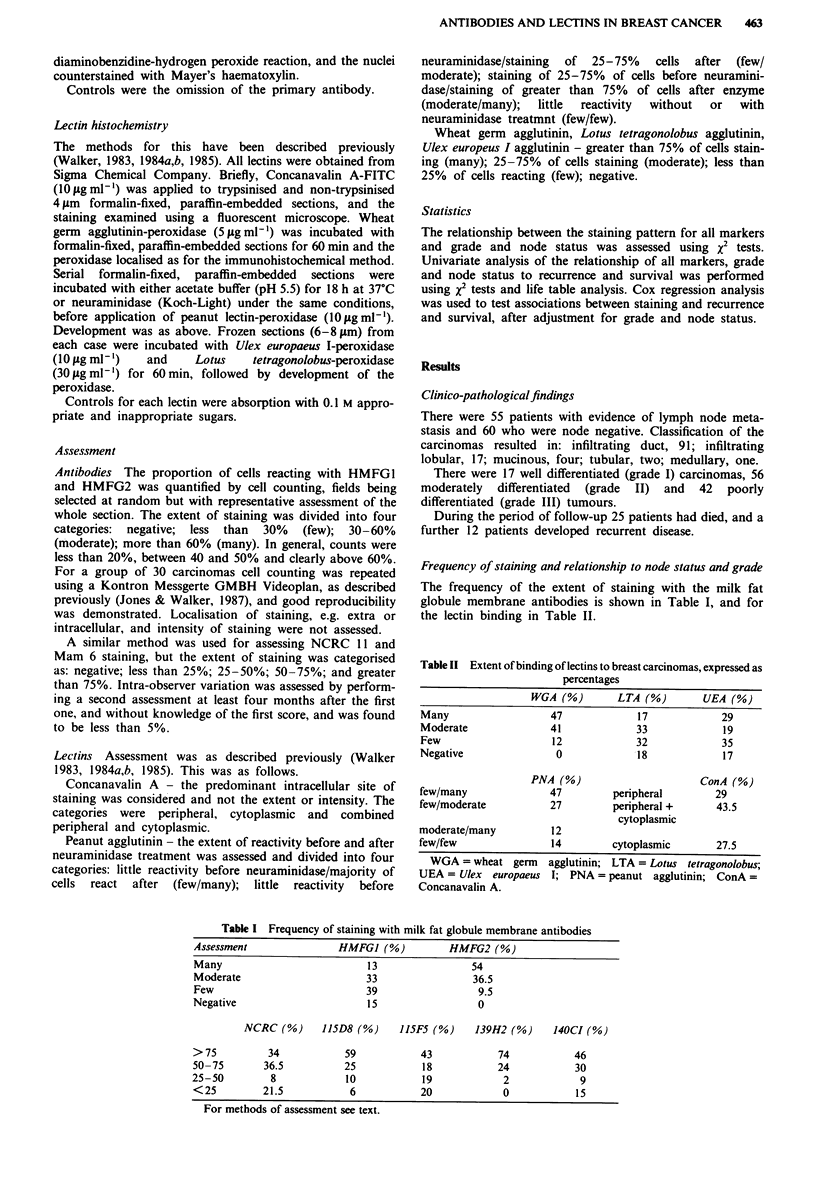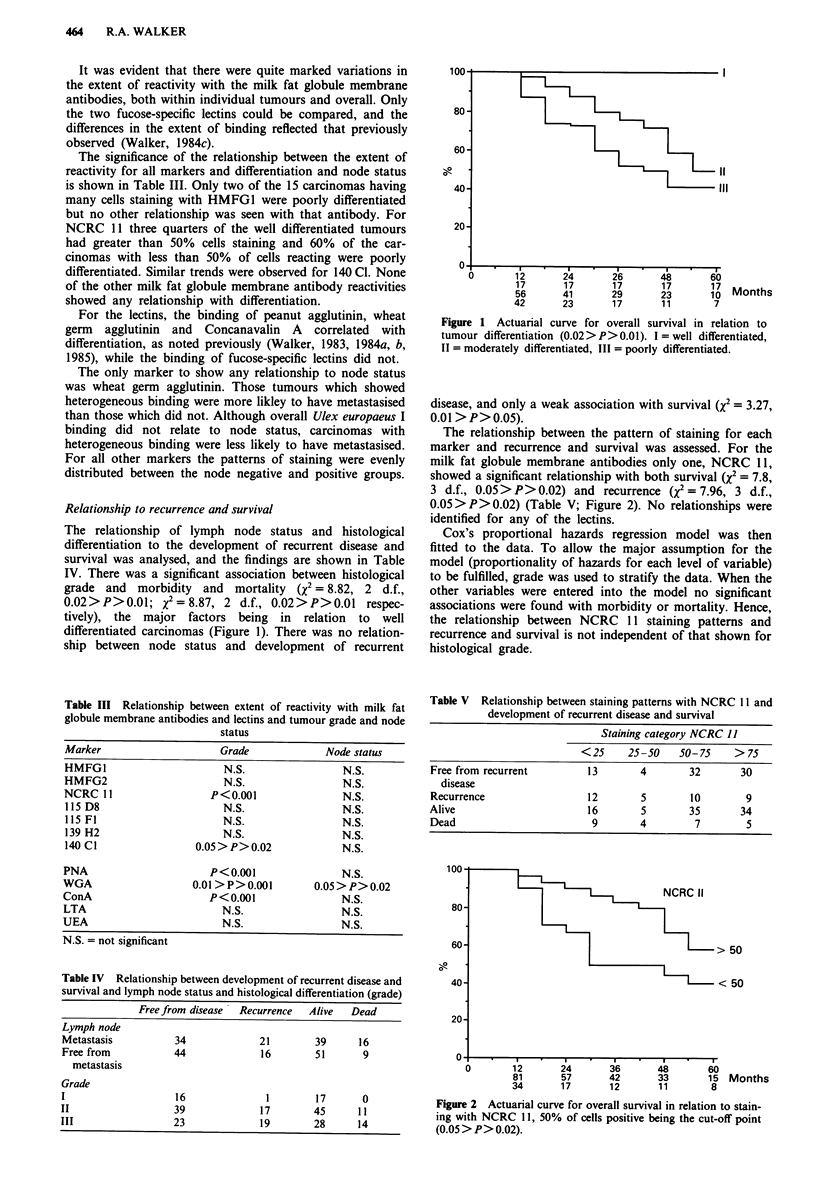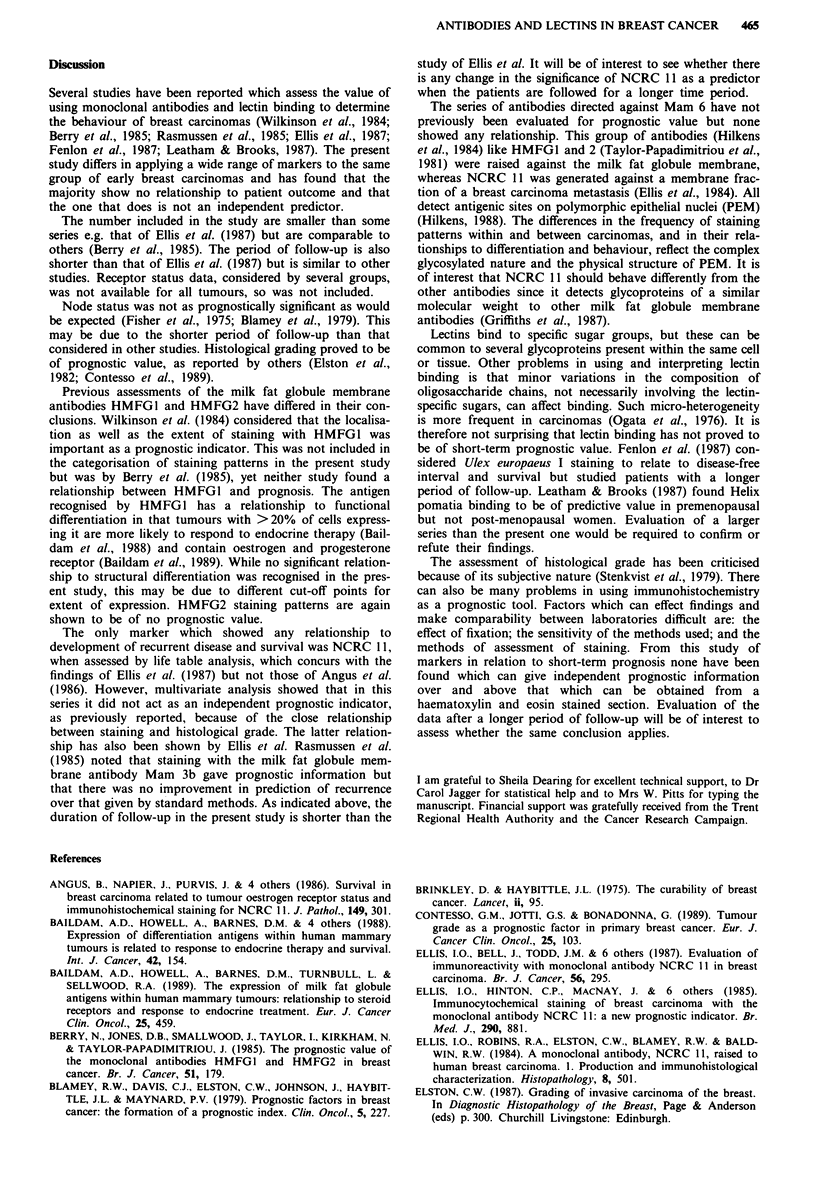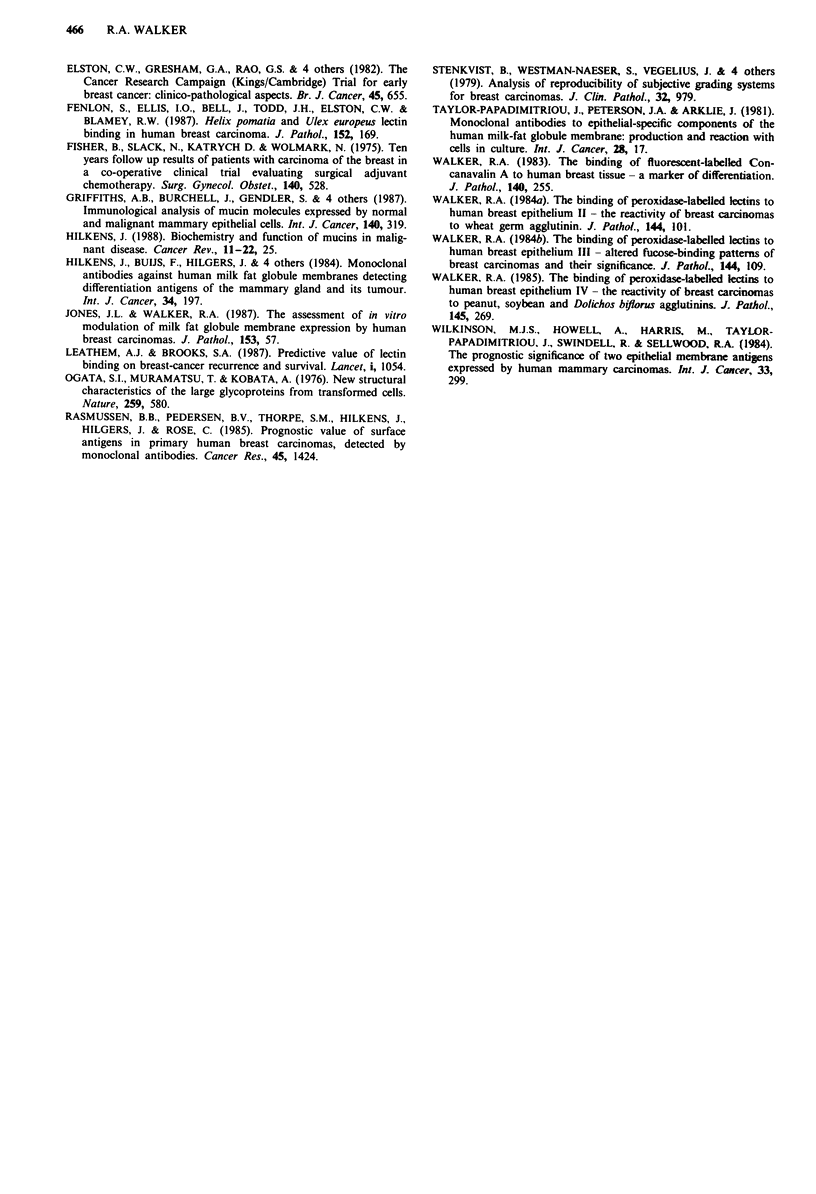# Assessment of milk fat globule membrane antibodies and lectins as markers of short-term prognosis in breast cancer.

**DOI:** 10.1038/bjc.1990.319

**Published:** 1990-09

**Authors:** R. A. Walker

**Affiliations:** Department of Pathology, University of Leicester, Leicester Royal Infirmary, UK.

## Abstract

The milk fat globule membrane antibodies HMFG1, HMFG2, NCRC 11 and four of the Mam 6 series, and the lectins peanut agglutinin, wheat germ agglutinin, Concanavalin A, Lotus tetragonolobus and Ulex europaeus I have been applied to 115 stage I and II breast carcinomas (median follow up = 36 months) to assess their value as prognostic markers. Of the milk fat globule membrane antibodies only NCRC 11 staining showed a relationship to development of recurrent disease and overall survival, but this did not act as an independent indicator over and above that provided by histological grade. None of the lectins gave prognostic information, including those whose binding related to node status or grade. It is concluded that for short-term prognosis none of the markers can given independent prognostic information over and above that provided by histological evaluation.


 © Macmillan Press Ltd., 1990

### Assessment of milk fat globule membrane antibodies and lectins as markers of short-term prognosis in breast cancer

R.A. Walker

*Department of Pathology, University of Leicester, Clinical Sciences Building, Leicester Royal Infirmary, PO Box 65, Leicester LE2 7LX, UK.*

**Summary** The milk fat globule membrane antibodies HMFG1, HMFG2, NCRC 11 and four of the Mam 6 series, and the lectins peanut agglutinin, wheat germ agglutinin, Concanavalin A, *Lotus tetragonolobus* and *Ulex europaeus* I have been applied to 115 stage I and II breast carcinomas (median follow up = 36 months) to assess their value as prognostic markers. Of the milk fat globule membrane antibodies only NCRC 11 staining showed a relationship to development of recurrent disease and overall survival, but this did not act as an independent indicator over and above that provided by histological grade. None of the lectins gave prognostic information, including those whose binding related to node status or grade. It is concluded that for short-term prognosis none of the markers can given independent prognostic information over and above that provided by histological evaluation.

The biological behaviour of breast carcinoma can vary considerably such that there are patients with a very poor prognosis (Blamey *et al.*, 1979) and those who remain free from disease for over 20 years (Brinkley & Haybittle, 1975). Factors such as the extent of spread (both nodal and distant) at the time of presentation are important in predicting behaviour, and this forms the basis of staging systems. However, changes in surgical practice with a reduction in axillary node sampling, plus a need to subdivide node negative early stage patients into poor or good risk categories means that there is a need to maximise the amount of prognostic information which can be obtained from the primary tumour.

The immunohistochemical assessment of primary tumours for the expression of various markers of potential prognostic value is one such approach. The use of antibodies to different components of the milk fat globule membrane has resulted in conflicting reports. Wilkinson *et al.* (1984) concluded that the antibody HMFG1 could be useful as a prognostic indicator, but Berry *et al.* (1985) found no relationship between the extent of staining with HMFG1 and HMFG2 and survival. Ellis *et al.* (1985, 1987) used the monoclonal antibody NCRC 11, which although raised against a breast carcinoma metastasis detects high molecular weight glycoproteins similar to those recognised by antibodies raised against the milk fat globule membrane. They found a clear relationship between immunoreactivity of carcinomas and the clinical course of the disease. Angus *et al.* (1986), however, using the same antibody, did not find a statistically significant relationship with prognosis. Direct comparison of the staining obtained with HMFG1, HMFG2 and NCRC 11 antibodies for the same group of carcinomas and their relationship to prognosis has not been published.

Another approach which has been evaluated for its prognostic value has been the binding of lectins to primary tumours. Greater reactivity of carcinomas with *Helix pomatia* was reported as relating to poorer survival (Fenlon *et al.*, 1987; Leathem & Brooks, 1987) as did *Ulex europaeus* I binding (Fenlon *et al.*, 1987).

In the present study a group of early (stage I and II) carcinomas have been studied for the expression of milk fat globule membrane antigens as determined by antibodies HMFG1, HMFG2, NCRC 11 and four against the Mam 6 antigen (Hilkens *et al.*, 1984), and for binding of the lectins Concanavalin A, peanut agglutinin, wheat germ agglutinin, *Lotus tetragonolobus* and *Ulex europaeus* I (Walker, 1983, 1984*a*,*b*, 1985), and the findings related to short-term recurrence and survival.

Received 20 November 1989; and in revised form 26 February 1990.

### Materials and methods

### *Patients*

Primary breast carcinomas excised from 115 patients between May 1981 and June 1986 were studied. Criteria for inclusion were: knowledge of lymph node status; tumour less than 5 cm in size; no clinical or pathological evidence of skin or muscle infiltration; no evidence of distant metastasis; no previous history of breast carcinoma and no subsequent development of a second primary tumour.

The primary treatment for the majority of patients was mastectomy with axillary dissection. A small number underwent lumpectomy and radiotherapy, with axillary node sampling. All patients with axillary lymph node metastasis received a course of radiotherapy. A small number (<10) were given adjuvant chemotherapy. Clinical follow-up was from 12 to 82 months with a median follow-up period of 36 months.

### *Tissues and histology*

All cases were received immediately after resection. Samples from each were frozen in isopentane cooled in liquid nitrogen, and stored in liquid nitrogen vapour phase. A parallel block was fixed in 4% formaldehyde in saline for 18-36 h and processed through to paraffin wax.

Haematoxylin and eosin stained sections were examined for classification using WHO criteria, and graded for differentiation using a modification of the Broom & Richardson grading system (Elston, 1987).

### *Immunohistochemistry*

The mouse monoclonal antibodies HMFG1 and HMFG2 were purchased from Oxoid Ltd. HMFG2 was also received as a gift from Dr J. Taylor-Papadimitriou. NCRC 11 was a gift of Dr I.O. Ellis, and the Mam 6 antibodies (115 D8, 115 F5, 139 H2 and 140 Cl) a gift of Dr J. Hilkens.

Sections (4 μm) were cut from each formalin-fixed, paraffin-embedded block. For all antibodies parallel sections were incubated with 0.1% trypsin (Difco 1:250) in 0.12% calcium chloride pH 7.8 at 37°C for 30 min or buffer. After washing the antibodies were applied to both trypsinised and non-trypsinised sections diluted in Tris-buffered saline pH 7.6 as follows: HMFG1, 1:10; HMFG2, 1:10; NCRC 11, 1:10; Mam 6 series, 1:100. The sections were incubated at 4°C for 18 h, followed by rinsing and washing in Tris-buffered saline. Peroxidase conjugated rabbit anti mouse immunoglobulin antiserum (Dako Ltd) was applied for 30 min. After rinsing and washing the peroxidase was localised by the diaminobenzidine-hydrogen peroxide reaction, and the nuclei counterstained with Mayer's haematoxylin.

Controls were the omission of the primary antibody.

*Lectin histochemistry*

The methods for this have been described previously (Walker, 1983, 1984*a*,*b*, 1985). All lectins were obtained from Sigma Chemical Company. Briefly, Concanavalin A-FITC ($10 \mu g ml^{-1}$) was applied to trypsinised and non-trypsinised $4 \mu m$ formalin-fixed, paraffin-embedded sections, and the staining examined using a fluorescent microscope. Wheat germ agglutinin-peroxidase ($5 \mu g ml^{-1}$) was incubated with formalin-fixed, paraffin-embedded sections for 60 min and the peroxidase localised as for the immunohistochemical method. Serial formalin-fixed, paraffin-embedded sections were incubated with either acetate buffer (pH 5.5) for 18 h at 37°C or neuraminidase (Koch-Light) under the same conditions, before application of peanut lectin-peroxidase ($10 \mu g ml^{-1}$). Development was as above. Frozen sections (6–8 $\mu m$) from each case were incubated with *Ulex europaeus* I-peroxidase ($10 \mu g ml^{-1}$) and *Lotus tetragonolobus*-peroxidase ($30 \mu g ml^{-1}$) for 60 min, followed by development of the peroxidase.

Controls for each lectin were absorption with 0.1 M appropriate and inappropriate sugars.

*Assessment*

*Antibodies* The proportion of cells reacting with HMFG1 and HMFG2 was quantified by cell counting, fields being selected at random but with representative assessment of the whole section. The extent of staining was divided into four categories: negative; less than 30% (few); 30–60% (moderate); more than 60% (many). In general, counts were less than 20%, between 40 and 50% and clearly above 60%. For a group of 30 carcinomas cell counting was repeated using a Kontron Messgerte GMBH Videoplan, as described previously (Jones & Walker, 1987), and good reproducibility was demonstrated. Localisation of staining, e.g. extra or intracellular, and intensity of staining were not assessed.

A similar method was used for assessing NCRC 11 and Mam 6 staining, but the extent of staining was categorised as: negative; less than 25%; 25–50%; 50–75%; and greater than 75%. Intra-observer variation was assessed by performing a second assessment at least four months after the first one, and without knowledge of the first score, and was found to be less than 5%.

*Lectins* Assessment was as described previously (Walker 1983, 1984*a*,*b*, 1985). This was as follows.

Concanavalin A – the predominant intracellular site of staining was considered and not the extent or intensity. The categories were peripheral, cytoplasmic and combined peripheral and cytoplasmic.

Peanut agglutinin – the extent of reactivity before and after neuraminidase treatment was assessed and divided into four categories: little reactivity before neuraminidase/majority of cells react after (few/many); little reactivity before neuraminidase/staining of 25–75% cells after (few/moderate); staining of 25–75% of cells before neuraminidase/staining of greater than 75% of cells after enzyme (moderate/many); little reactivity without or with neuraminidase treatmnt (few/few).

Wheat germ agglutinin, *Lotus tetragonolobus* agglutinin, *Ulex europeus I* agglutinin – greater than 75% of cells staining (many); 25–75% of cells staining (moderate); less than 25% of cells reacting (few); negative.

*Statistics*

The relationship between the staining pattern for all markers and grade and node status was assessed using $\chi^2$ tests. Univariate analysis of the relationship of all markers, grade and node status to recurrence and survival was performed using $\chi^2$ tests and life table analysis. Cox regression analysis was used to test associations between staining and recurrence and survival, after adjustment for grade and node status.

### Results

*Clinico-pathological findings*

There were 55 patients with evidence of lymph node metastasis and 60 who were node negative. Classification of the carcinomas resulted in: infiltrating duct, 91; infiltrating lobular, 17; mucinous, four; tubular, two; medullary, one.

There were 17 well differentiated (grade I) carcinomas, 56 moderately differentiated (grade II) and 42 poorly differentiated (grade III) tumours.

During the period of follow-up 25 patients had died, and a further 12 patients developed recurrent disease.

*Frequency of staining and relationship to node status and grade*

The frequency of the extent of staining with the milk fat globule membrane antibodies is shown in Table I, and for the lectin binding in Table II.

**Table II** Extent of binding of lectins to breast carcinomas, expressed as percentages

| | *WGA (%)* | *LTA (%)* | *UEA (%)* |
|---|---|---|---|
| Many | 47 | 17 | 29 |
| Moderate | 41 | 33 | 19 |
| Few | 12 | 32 | 35 |
| Negative | 0 | 18 | 17 |

| | *PNA (%)* | | *ConA (%)* |
|---|---|---|---|
| few/many | 47 | peripheral | 29 |
| few/moderate | 27 | peripheral + cytoplasmic | 43.5 |
| moderate/many | 12 | | |
| few/few | 14 | cytoplasmic | 27.5 |

WGA = wheat germ agglutinin; LTA = *Lotus tetragonolobus*; UEA = *Ulex europaeus* I; PNA = peanut agglutinin; ConA = Concanavalin A.

**Table I** Frequency of staining with milk fat globule membrane antibodies

| *Assessment* | *HMFG1 (%)* | *HMFG2 (%)* |
|---|---|---|
| Many | 13 | 54 |
| Moderate | 33 | 36.5 |
| Few | 39 | 9.5 |
| Negative | 15 | 0 |

| | *NCRC (%)* | *115D8 (%)* | *115F5 (%)* | *139H2 (%)* | *140CI (%)* |
|---|---|---|---|---|---|
| >75 | 34 | 59 | 43 | 74 | 46 |
| 50–75 | 36.5 | 25 | 18 | 24 | 30 |
| 25–50 | 8 | 10 | 19 | 2 | 9 |
| <25 | 21.5 | 6 | 20 | 0 | 15 |

For methods of assessment see text.

It was evident that there were quite marked variations in the extent of reactivity with the milk fat globule membrane antibodies, both within individual tumours and overall. Only the two fucose-specific lectins could be compared, and the differences in the extent of binding reflected that previously observed (Walker, 1984*c*).

The significance of the relationship between the extent of reactivity for all markers and differentiation and node status is shown in Table III. Only two of the 15 carcinomas having many cells staining with HMFG1 were poorly differentiated but no other relationship was seen with that antibody. For NCRC 11 three quarters of the well differentiated tumours had greater than 50% cells staining and 60% of the carcinomas with less than 50% of cells reacting were poorly differentiated. Similar trends were observed for 140 C1. None of the other milk fat globule membrane antibody reactivities showed any relationship with differentiation.

For the lectins, the binding of peanut agglutinin, wheat germ agglutinin and Concanavalin A correlated with differentiation, as noted previously (Walker, 1983, 1984*a*, *b*, 1985), while the binding of fucose-specific lectins did not.

The only marker to show any relationship to node status was wheat germ agglutinin. Those tumours which showed heterogeneous binding were more likley to have metastasised than those which did not. Although overall *Ulex europaeus* I binding did not relate to node status, carcinomas with heterogeneous binding were less likely to have metastasised. For all other markers the patterns of staining were evenly distributed between the node negative and positive groups.

*Relationship to recurrence and survival*

The relationship of lymph node status and histological differentiation to the development of recurrent disease and survival was analysed, and the findings are shown in Table IV. There was a significant association between histological grade and morbidity and mortality ($\chi^2 = 8.82$, 2 d.f., $0.02 > P > 0.01$; $\chi^2 = 8.87$, 2 d.f., $0.02 > P > 0.01$ respectively), the major factors being in relation to well differentiated carcinomas (Figure 1). There was no relationship between node status and development of recurrent disease, and only a weak association with survival ($\chi^2 = 3.27$, $0.01 > P > 0.05$).

**Figure 1** Actuarial curve for overall survival in relation to tumour differentiation ($0.02 > P > 0.01$). I = well differentiated, II = moderately differentiated, III = poorly differentiated.

The relationship between the pattern of staining for each marker and recurrence and survival was assessed. For the milk fat globule membrane antibodies only one, NCRC 11, showed a significant relationship with both survival ($\chi^2 = 7.8$, 3 d.f., $0.05 > P > 0.02$) and recurrence ($\chi^2 = 7.96$, 3 d.f., $0.05 > P > 0.02$) (Table V; Figure 2). No relationships were identified for any of the lectins.

Cox's proportional hazards regression model was then fitted to the data. To allow the major assumption for the model (proportionality of hazards for each level of variable) to be fulfilled, grade was used to stratify the data. When the other variables were entered into the model no significant associations were found with morbidity or mortality. Hence, the relationship between NCRC 11 staining patterns and recurrence and survival is not independent of that shown for histological grade.

**Table III** Relationship between extent of reactivity with milk fat globule membrane antibodies and lectins and tumour grade and node status

| *Marker* | *Grade* | *Node status* |
|---|---|---|
| HMFG1 | N.S. | N.S. |
| HMFG2 | N.S. | N.S. |
| NCRC 11 | $P < 0.001$ | N.S. |
| 115 D8 | N.S. | N.S. |
| 115 F1 | N.S. | N.S. |
| 139 H2 | N.S. | N.S. |
| 140 C1 | $0.05 > P > 0.02$ | N.S. |
| PNA | $P < 0.001$ | N.S. |
| WGA | $0.01 > P > 0.001$ | $0.05 > P > 0.02$ |
| ConA | $P < 0.001$ | N.S. |
| LTA | N.S. | N.S. |
| UEA | N.S. | N.S. |

N.S. = not significant

**Table IV** Relationship between development of recurrent disease and survival and lymph node status and histological differentiation (grade)

| | *Free from disease* | *Recurrence* | *Alive* | *Dead* |
|---|---|---|---|---|
| *Lymph node* | | | | |
| Metastasis | 34 | 21 | 39 | 16 |
| Free from metastasis | 44 | 16 | 51 | 9 |
| *Grade* | | | | |
| I | 16 | 1 | 17 | 0 |
| II | 39 | 17 | 45 | 11 |
| III | 23 | 19 | 28 | 14 |

**Table V** Relationship between staining patterns with NCRC 11 and development of recurrent disease and survival

| | *Staining category NCRC 11* | | | |
|---|---|---|---|---|
| | *<25* | *25–50* | *50–75* | *>75* |
| Free from recurrent disease | 13 | 4 | 32 | 30 |
| Recurrence | 12 | 5 | 10 | 9 |
| Alive | 16 | 5 | 35 | 34 |
| Dead | 9 | 4 | 7 | 5 |

**Figure 2** Actuarial curve for overall survival in relation to staining with NCRC 11, 50% of cells positive being the cut-off point ($0.05 > P > 0.02$).

### Discussion

Several studies have been reported which assess the value of using monoclonal antibodies and lectin binding to determine the behaviour of breast carcinomas (Wilkinson *et al.*, 1984; Berry *et al.*, 1985; Rasmussen *et al.*, 1985; Ellis *et al.*, 1987; Fenlon *et al.*, 1987; Leatham & Brooks, 1987). The present study differs in applying a wide range of markers to the same group of early breast carcinomas and has found that the majority show no relationship to patient outcome and that the one that does is not an independent predictor.

The number included in the study are smaller than some series e.g. that of Ellis *et al.* (1987) but are comparable to others (Berry *et al.*, 1985). The period of follow-up is also shorter than that of Ellis *et al.* (1987) but is similar to other studies. Receptor status data, considered by several groups, was not available for all tumours, so was not included.

Node status was not as prognostically significant as would be expected (Fisher *et al.*, 1975; Blamey *et al.*, 1979). This may be due to the shorter period of follow-up than that considered in other studies. Histological grading proved to be of prognostic value, as reported by others (Elston *et al.*, 1982; Contesso *et al.*, 1989).

Previous assessments of the milk fat globule membrane antibodies HMFG1 and HMFG2 have differed in their conclusions. Wilkinson *et al.* (1984) considered that the localisation as well as the extent of staining with HMFG1 was important as a prognostic indicator. This was not included in the categorisation of staining patterns in the present study but was by Berry *et al.* (1985), yet neither study found a relationship between HMFG1 and prognosis. The antigen recognised by HMFG1 has a relationship to functional differentiation in that tumours with $>20\%$ of cells expressing it are more likely to respond to endocrine therapy (Baildam *et al.*, 1988) and contain oestrogen and progesterone receptor (Baildam *et al.*, 1989). While no significant relationship to structural differentiation was recognised in the present study, this may be due to different cut-off points for extent of expression. HMFG2 staining patterns are again shown to be of no prognostic value.

The only marker which showed any relationship to development of recurrent disease and survival was NCRC 11, when assessed by life table analysis, which concurs with the findings of Ellis *et al.* (1987) but not those of Angus *et al.* (1986). However, multivariate analysis showed that in this series it did not act as an independent prognostic indicator, as previously reported, because of the close relationship between staining and histological grade. The latter relationship has also been shown by Ellis *et al.* Rasmussen *et al.* (1985) noted that staining with the milk fat globule membrane antibody Mam 3b gave prognostic information but that there was no improvement in prediction of recurrence over that given by standard methods. As indicated above, the duration of follow-up in the present study is shorter than the study of Ellis *et al.* It will be of interest to see whether there is any change in the significance of NCRC 11 as a predictor when the patients are followed for a longer time period.

The series of antibodies directed against Mam 6 have not previously been evaluated for prognostic value but none showed any relationship. This group of antibodies (Hilkens *et al.*, 1984) like HMFG1 and 2 (Taylor-Papadimitriou *et al.*, 1981) were raised against the milk fat globule membrane, whereas NCRC 11 was generated against a membrane fraction of a breast carcinoma metastasis (Ellis *et al.*, 1984). All detect antigenic sites on polymorphic epithelial nuclei (PEM) (Hilkens, 1988). The differences in the frequency of staining patterns within and between carcinomas, and in their relationships to differentiation and behaviour, reflect the complex glycosylated nature and the physical structure of PEM. It is of interest that NCRC 11 should behave differently from the other antibodies since it detects glycoproteins of a similar molecular weight to other milk fat globule membrane antibodies (Griffiths *et al.*, 1987).

Lectins bind to specific sugar groups, but these can be common to several glycoproteins present within the same cell or tissue. Other problems in using and interpreting lectin binding is that minor variations in the composition of oligosaccharide chains, not necessarily involving the lectin-specific sugars, can affect binding. Such micro-heterogeneity is more frequent in carcinomas (Ogata *et al.*, 1976). It is therefore not surprising that lectin binding has not proved to be of short-term prognostic value. Fenlon *et al.* (1987) considered *Ulex europaeus* I staining to relate to disease-free interval and survival but studied patients with a longer period of follow-up. Leatham & Brooks (1987) found Helix pomatia binding to be of predictive value in premenopausal but not post-menopausal women. Evaluation of a larger series than the present one would be required to confirm or refute their findings.

The assessment of histological grade has been criticised because of its subjective nature (Stenkvist *et al.*, 1979). There can also be many problems in using immunohistochemistry as a prognostic tool. Factors which can effect findings and make comparability between laboratories difficult are: the effect of fixation; the sensitivity of the methods used; and the methods of assessment of staining. From this study of markers in relation to short-term prognosis none have been found which can give independent prognostic information over and above that which can be obtained from a haematoxylin and eosin stained section. Evaluation of the data after a longer period of follow-up will be of interest to assess whether the same conclusion applies.

I am grateful to Sheila Dearing for excellent technical support, to Dr Carol Jagger for statistical help and to Mrs W. Pitts for typing the manuscript. Financial support was gratefully received from the Trent Regional Health Authority and the Cancer Research Campaign.

### References

ANGUS, B., NAPIER, J., PURVIS, J. & 4 others (1986). Survival in breast carcinoma related to tumour oestrogen receptor status and immunohistochemical staining for NCRC 11. *J. Pathol.*, **149**, 301.

BAILDAM, A.D., HOWELL, A., BARNES, D.M. & 4 others (1988). Expression of differentiation antigens within human mammary tumours is related to response to endocrine therapy and survival. *Int. J. Cancer*, **42**, 154.

BAILDAM, A.D., HOWELL, A., BARNES, D.M., TURNBULL, L. & SELLWOOD, R.A. (1989). The expression of milk fat globule antigens within human mammary tumours: relationship to steroid receptors and response to endocrine treatment. *Eur. J. Cancer Clin. Oncol.*, **25**, 459.

BERRY, N., JONES, D.B., SMALLWOOD, J., TAYLOR, I., KIRKHAM, N. & TAYLOR-PAPADIMITRIOU, J. (1985). The prognostic value of the monoclonal antibodies HMFG1 and HMFG2 in breast cancer. *Br. J. Cancer*, **51**, 179.

BLAMEY, R.W., DAVIS, C.J., ELSTON, C.W., JOHNSON, J., HAYBITTLE, J.L. & MAYNARD, P.V. (1979). Prognostic factors in breast cancer: the formation of a prognostic index. *Clin. Oncol.*, **5**, 227.

BRINKLEY, D. & HAYBITTLE, J.L. (1975). The curability of breast cancer. *Lancet*, **ii**, 95.

CONTESSO, G.M., JOTTI, G.S. & BONADONNA, G. (1989). Tumour grade as a prognostic factor in primary breast cancer. *Eur. J. Cancer Clin. Oncol.*, **25**, 103.

ELLIS, I.O., BELL, J., TODD, J.M. & 6 others (1987). Evaluation of immunoreactivity with monoclonal antibody NCRC 11 in breast carcinoma. *Br. J. Cancer*, **56**, 295.

ELLIS, I.O., HINTON, C.P., MACNAY, J. & 6 others (1985). Immunocytochemical staining of breast carcinoma with the monoclonal antibody NCRC 11: a new prognostic indicator. *Br. Med. J.*, **290**, 881.

ELLIS, I.O., ROBINS, R.A., ELSTON, C.W., BLAMEY, R.W. & BALDWIN, R.W. (1984). A monoclonal antibody, NCRC 11, raised to human breast carcinoma. 1. Production and immunohistological characterization. *Histopathology*, **8**, 501.

ELSTON, C.W. (1987). Grading of invasive carcinoma of the breast. In *Diagnostic Histopathology of the Breast*, Page & Anderson (eds) p. 300. Churchill Livingstone: Edinburgh.

ELSTON, C.W., GRESHAM, G.A., RAO, G.S. & 4 others (1982). The Cancer Research Campaign (Kings/Cambridge) Trial for early breast cancer: clinico-pathological aspects. *Br. J. Cancer*, **45**, 655.

FENLON, S., ELLIS, I.O., BELL, J., TODD, J.H., ELSTON, C.W. & BLAMEY, R.W. (1987). *Helix pomatia* and *Ulex europeus* lectin binding in human breast carcinoma. *J. Pathol.*, **152**, 169.

FISHER, B., SLACK, N., KATRYCH D. & WOLMARK, N. (1975). Ten years follow up results of patients with carcinoma of the breast in a co-operative clinical trial evaluating surgical adjuvant chemotherapy. *Surg. Gynecol. Obstet.*, **140**, 528.

GRIFFITHS, A.B., BURCHELL, J., GENDLER, S. & 4 others (1987). Immunological analysis of mucin molecules expressed by normal and malignant mammary epithelial cells. *Int. J. Cancer*, **140**, 319.

HILKENS, J. (1988). Biochemistry and function of mucins in malignant disease. *Cancer Rev.*, **11–22**, 25.

HILKENS, J., BUIJS, F., HILGERS, J. & 4 others (1984). Monoclonal antibodies against human milk fat globule membranes detecting differentiation antigens of the mammary gland and its tumour. *Int. J. Cancer*, **34**, 197.

JONES, J.L. & WALKER, R.A. (1987). The assessment of *in vitro* modulation of milk fat globule membrane expression by human breast carcinomas. *J. Pathol.*, **153**, 57.

LEATHEM, A.J. & BROOKS, S.A. (1987). Predictive value of lectin binding on breast-cancer recurrence and survival. *Lancet*, **i**, 1054.

OGATA, S.I., MURAMATSU, T. & KOBATA, A. (1976). New structural characteristics of the large glycoproteins from transformed cells. *Nature*, **259**, 580.

RASMUSSEN, B.B., PEDERSEN, B.V., THORPE, S.M., HILKENS, J., HILGERS, J. & ROSE, C. (1985). Prognostic value of surface antigens in primary human breast carcinomas, detected by monoclonal antibodies. *Cancer Res.*, **45**, 1424.

STENKVIST, B., WESTMAN-NAESER, S., VEGELIUS, J. & 4 others (1979). Analysis of reproducibility of subjective grading systems for breast carcinomas. *J. Clin. Pathol.*, **32**, 979.

TAYLOR-PAPADIMITRIOU, J., PETERSON, J.A. & ARKLIE, J. (1981). Monoclonal antibodies to epithelial-specific components of the human milk-fat globule membrane: production and reaction with cells in culture. *Int. J. Cancer*, **28**, 17.

WALKER, R.A. (1983). The binding of fluorescent-labelled Concanavalin A to human breast tissue – a marker of differentiation. *J. Pathol.*, **140**, 255.

WALKER, R.A. (1984*a*). The binding of peroxidase-labelled lectins to human breast epithelium II – the reactivity of breast carcinomas to wheat germ agglutinin. *J. Pathol.*, **144**, 101.

WALKER, R.A. (1984*b*). The binding of peroxidase-labelled lectins to human breast epithelium III – altered fucose-binding patterns of breast carcinomas and their significance. *J. Pathol.*, **144**, 109.

WALKER, R.A. (1985). The binding of peroxidase-labelled lectins to human breast epithelium IV – the reactivity of breast carcinomas to peanut, soybean and *Dolichos biflorus* agglutinins. *J. Pathol.*, **145**, 269.

WILKINSON, M.J.S., HOWELL, A., HARRIS, M., TAYLOR-PAPADIMITRIOU, J., SWINDELL, R. & SELLWOOD, R.A. (1984). The prognostic significance of two epithelial membrane antigens expressed by human mammary carcinomas. *Int. J. Cancer*, **33**, 299.